# Supplementary material for: Distant relatives of a eukaryotic cell-specific toxin family evolved a complement-like mechanism to kill bacteria
Source: Nat Commun. 2024 Jun 12;15:5028. doi: 10.1038/s41467-024-49103-5 (PMC11169675; doi:10.1038/s41467-024-49103-5)
Supplement: Supplementary file 1 — Supplementary Information [file 41467_2024_49103_MOESM1_ESM.pdf]

Supplementary Information

Supplementary Fig. 1. *Representative electron density used to model the EaCDCL*^{S} *crystal structure.* (a) Modeled structure of EaCDCL^{S} focused on the novel loop region cleaved during activation (shown as blue sticks) and corresponding electron density map (grey mesh; 2F_o-F_c map shown at 1.0σ). (b) As per (a) but focused on core β-sheet of domain 3 (shown as yellow sticks). (c) As per (a) but focused on transmembrane hairpin 2 (TMH2) helical bundle (shown as green sticks).

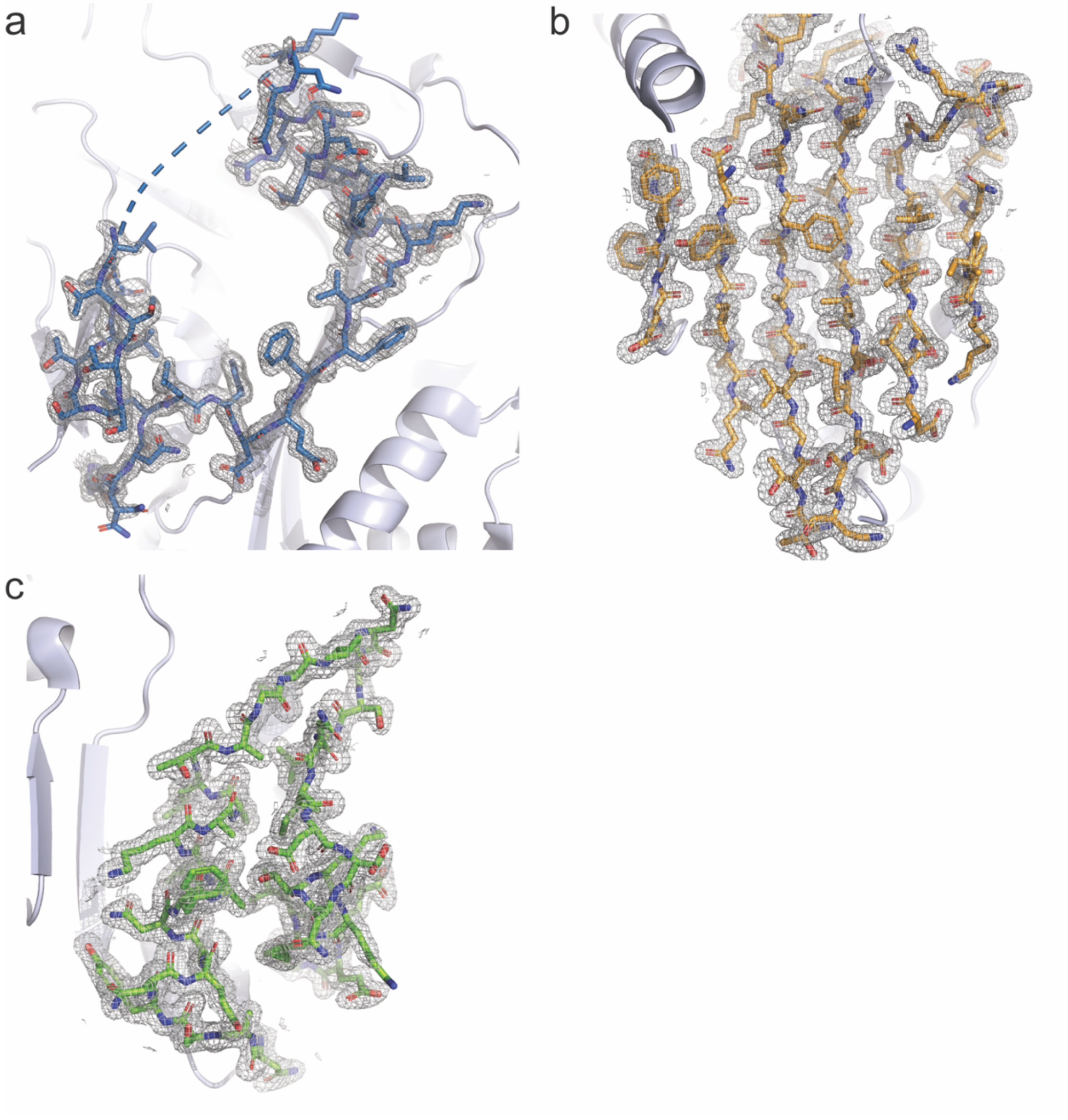

Supplementary Fig. 2. *CLUSTAL O (1.2.4)[1] multiple sequence alignment of the primary structures of PFO, EaCDCLs and BfCDCLs.* Alignment of PFO with the *E. anophelis* and *B. fragilis* CDCLs. Note, as shown before that the YGR and GG residues are conserved across all 4 proteins, except that the BfCDCL-contains an NG instead of the GG pair. The conserved TL cholesterol binding motif of the CDCs is shown in bold and underline. The CDC signature motif of ECTGLAWEWWR is shown in bold italics. The PFO binding domain (domain 4 in Fig. 2) and the CDCL putative binding domains are italicized. The residues that consititute the β-tongue structure of PFO are highlighted in green. Shown in grey highlight are the residues that are not imaged in the crystal structures of EaCDCL^{L} and EaCDCL^{S}. Bold red residues are the *in vitro* and *in vivo* proteolytic activation sites identified for each protein.

```
PFO           MIRFKKTKLIASIAMALCL-FSQPVISFSKDITDKNQ-----------------------   36
EaCDCL-L      MRIY------SLFSLALAVTSSVIITSCATDNLPDRS--SV--TNNMARVELPVINITSF   50
BfCDCL-L      MRIL------RYTL---FL-FAIGLISCEQEFDKKEQQSDCCSFQNLPKYPEQVVNINVH   50
EaCDCL-S      MKKL------LFPIVALAA-TSLTIISCRQDSEVNPL---------QVQNSSKVLNPNVT   44
BfCDCL-S      MKQY------VYLL---IL-PIMFLISCSDDTVEEMGGSN---HVLLERYPQEVINISMD   47
              *                       : *   :   .

PFO           --SIDSGISSLSYNRNEVLASNGDKIESFVPKEGKKAGNK-FIVVERQKRSLTTSP---V   90
EaCDCL-L      GTKP----SFLNIQKSASTKSL-NLIAENS---GD------------TETKEFESSESV-   89
BfCDCL-L      NYIPDMGDKVLSSPSHIHTRAS-EIISETLWDNGF------------GKTIHAQADESI-   96
EaCDCL-S      LPA-----NNLLYDEFFVSKES-KLIEDSRNNKRKTSKIASLNPYASTKAVLTTTSSTLT   98
BfCDCL-S      NPE-----SFASFNKTSVTRSS-ELLKDSVWSSGN------------GHTLIYETNEHIP   89
                      .              . : .                    .     :

PFO           DISIIDSVNDRTYPGALQLADKAFVENRPTILMVKRKPININIDLPGLKGENSIKVDDPT   150
EaCDCL-L      ---VLNHLNRYVFPGSLLMGNSI-QDLNYKPVFASLNPITVSLSIPAINQNTAITITNPS   145
BfCDCL-L      ---VLDNLNRYIYPGSILDGASV-ANQDYKTISVHYKPINVSVSFPAQKV--TGVLEKPS   150
EaCDCL-S      SDQIVVTVPQKTFIGGVYNSTTL-DNLDYTPISYPLDPITVSYSFPSDFI--VDTIERPS   155
BfCDCL-S      ----TQGLMRYIYLGSILQGGSI-EKQRFVPIVKPMDPITISYSFPARWV--TDIIAKPS   142
                     :    : *.:  . .   .     :    .**.:. .:*.        :  *:

PFO           YGKVSGAIDELVSKWNEKYSSTHTLPARTQYSESMVYSKSQ--------ISSALNVNAKV   202
EaCDCL-L      LSATRAAVYNYLKTADF----------T--QNGQLSYSIQQFSSYDELKVAFGSNVNSRN   193
BfCDCL-L      LSSCRQLVMDLMHQKGI-----------GQQSASVHFDIHRFTSYDELKMTFGSNANTSF   199
EaCDCL-S      LSSMRASVFKAMRAANF----------SGEQSLAFDYNIKQFSYYSELKIAFGSNVNIGK   205
BfCDCL-S      LSAQRQSLQNIMNKEGM----------TGKQLGSFTYNMRQFSYFEELKLAFGANVNIGG   192
               .     : . :   .                  .:.   :        :: . *.*

PFO           LENSLGVDFNA-----VANNEKKVMILAYKQIFYTVSADLPKNPSDLFDDSVTFNDLKQK   257
EaCDCL-L      L---FGKNSSSTNVEEGMVARQSGFYVKFYQTSFTLDMDVPNGS-------LVKDNNFD-   242
BfCDCL-L      L---FWGSSSSQQEHKERISKSSGLYIRFIQKYFTIDMDIPEKS-------FIE-----G   244
EaCDCL-S      I---FSIDIS---GSNNKIKRTTGVFAKFTQKNFTIDMDLPADG-------NIFKNNSDL   252
BfCDCL-S      L---LNIDVS---LDKGKIRKKTGLFAKIVQRNYTVDMDLPADG-------NILLNHDDM   239
              :   :  . .          . . .     *  :*:. *:*

PFO           GVSNEAPPLMVSNVAYGRTIYVKLETTSSSKDVQ----AAFKALIKNTDIKNSQQYKDIY   313
EaCDCL-L      --SEGIEPVYVSSISYGRMGILAIETNEKAEDAKRIINETFNKLFYKKQTNFSQEEKSFI   300
BfCDCL-L      SINAQYSPVYVSSIAYGRVGILTLETDEIYENAESIVKKAVNGFLYNKKEFLTVEEKGFF   304
EaCDCL-S      ALTNGKNPVYISSVTYGRLGIISIESNASYNEVNFALKAALTAGIVNGSLNIDSNSKKIL   312
BfCDCL-S      GSVGKYDPIYISSITYGRMALISIESSESYDKVRIALQAALQAKVVNGKLSFNLEQEKIL   299
                     *: :*.::***   : :*:    ....     :.   . : .     : : :

PFO           ENSSFTAVVLGGDAQEHNKVVTKDFDEIRKVIKDNATFSTKNPAYPISYTSVFLKDNSVA   373
EaCDCL-L      EGADFNLYLVGGDGSTASQ-SFKGYEAFVNHVSQ-GTFSKDQPGVPIFCSYSYLKDNSPV   358
BfCDCL-L      DEARMKVYVGGGNGDSGVK-TLTGFDDFIKFISEGGHFSAETPGKPIFCSFAYLSDHSPY   363
EaCDCL-S      EESDLSVYLVGGRGTDAVQ-VIKGFAGFSNFIVNGGQFTPEAPGVPIYFSASHASDNSVY   371
BfCDCL-S      KEAEVNVMVYNGEGEGTVK-TAKGWNEFQDFIIQGGRFSKDLPGDAIFYTASYLSDNSPF   358
              . : ..  : .* .    :   ..:  : . : : . *: . *.  *  :  . .*:*

PFO           AVHNKTDYIETTSTEYSKGKINLDHSGAYVAQFEVAWDEVSYDKEGNEVLTHKTWDGNYQ   429
EaCDCL-L      KTKFKFDIKRPP---LYVKLVKENMKDINFNDPDGGIYDNKKEAILKIYFYKNRSLVPTL   403
BfCDCL-L      KVKFKIDIDS-----DPVYARIEYRNLKNDSYVGRSLQREKIIGDVHLAFYADRTAKIPT   406
EaCDCL-S      YTTFTIDK----------------------------------------------------   379
BfCDCL-S      YSKFKIHLEN--------------------------------------------------   368
                  .               :.

PFO           DKTAHYSTVIPLEANARNIRIKARECTGLAWEWWRDVISEYDVPLTNNINVSIWGTTLYP   458
EaCDCL-L      PNPYINFKIREKKKKWQSIAPVYYSSLDQVPFNISERILTKQNTLQNIFATIQTQDNTEF   461
BfCDCL-L      VAPRYISFNIVEHSRHYLKARIPRTEKDDTAVEEFVKSNNSRGTELQLKHDLLLTEFVWT   450

PFO           GSSITYN-----------------------------------------------------   500
EaCDCL-L      SLISRIIRGGGRQNPVPAGFRAIEINDYELVEDSNYIIIKD-------------------   516
BfCDCL-L      HNNPRRTRYEQNTFRYSLSEGGFYKILPAINEEPKKWR----------------------   515
```

Supplementary Fig. 3. *Alphafold metrics for the BfCDCL^{L} and CDCL^{S} structures***.** (**a**) Shown in the first panel is the sequence coverage which is a graphical representation of the number of similar sequences from a multiple sequence alignment. It is evident the number similar sequences drops off dramatically at both the N- and C-terminal regions. In the second panel to the right is the predicted Local Distance Difference Test (pLDDT) for the *B. fragilis* CDCL^{L}, which reflects the confidence in the folded structure along the length of the protein.The amino terminal region exhibits two regions of pLDDT values <50% showing a low confidence in the structure. In the third panel is the Predicted Aligned Error (PAE) which estimates the relative position of the domain within the protein. The amino terminal region also exhibits a high PAE score suggestion the position of this region is uncertain. The X and Y axis of the PAE plots reflect the aminio acid number. (**b**) The same analyses are shown for BfCDCL^{S}. Similar to the BFCDCL^{L} the amino terminal region structure also exhibits low pLDDT and high PAE scores for its amino terminal region, which also suggests a low confidence in its structure and its relative position. Since the CDCL^{S} lacks an analogous binding region the sequence coverage ends at residue 350.

a

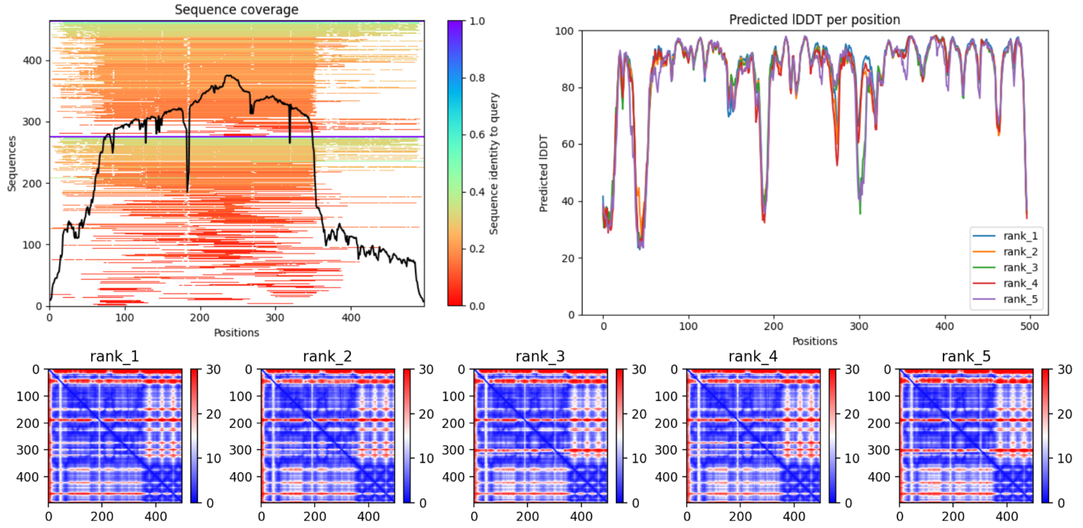

b

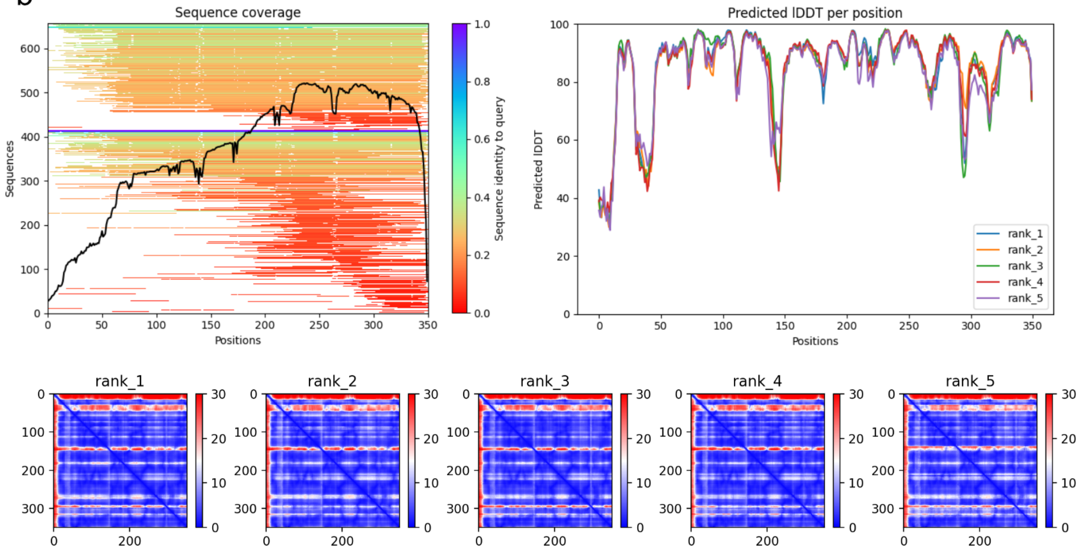

Supplementary Fig. 4. *CDCL oligomer gel.* Reactions were carried out using the following molar ratios for each reaction: 8.1 μM $CDCL^{L}$ (first, second, seventh, and eighth lanes), 24.4 μM $CDCL^{S}$ (third, fourth, ninth, and tenth lanes), 810 nM $CDCL^{L}$ and 24.4 μM $CDCL^{S}$ (fifth, sixth, eleventh, and twelfth lanes), and 8.9 PFO μM (thirteenth lane). All reactions contained liposomes at a concentration of 22 μM, with POPC liposomes used for all CDCL reactions and cholesterol-POPC liposomes used for the experiment with PFO. Trypsin from bovine pancreas (Sigma-Aldrich) was added at a 15:1 CDCL:trypsin ratio (w/w) and was carried out for 30 minutes in HEPES buffered saline (50mM HEPES, 100mM NaCl, pH 7.4) at a total volume of 21.5 μL. Each sample was quenched with a 4x SDS and DTT, heated to 95°C, and analyzed via SDS-AGE as previously described[2]. PFO, *Clostridium perfringens* CDC perfringolysin O

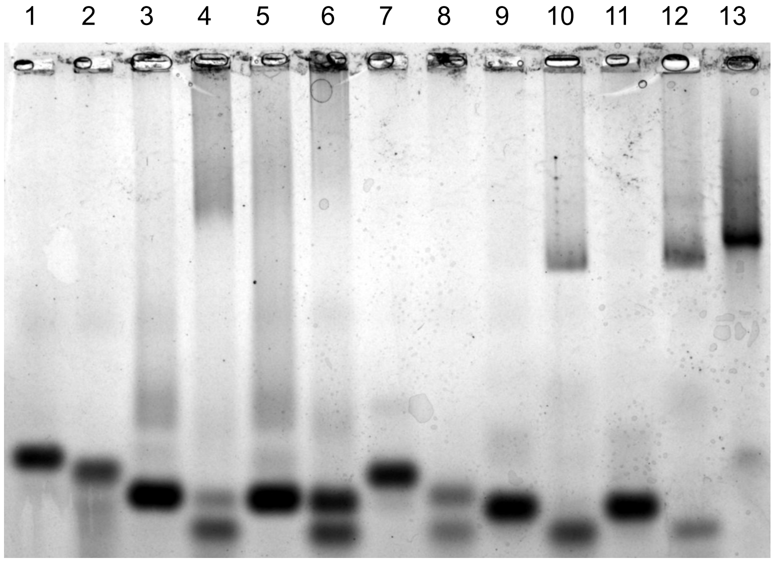

1. $EaCDCL^{L}$
2. $EaCDCL^{L}$ + Trypsin
3. $EaCDCL^{S}$
4. $EaCDCL^{S}$ + Trypsin
5. $EaCDCL^{L}$ + $EaCDCL^{S}$
6. $EaCDCL^{L}$ + $EaCDCL^{S}$ + Trypsin
7. $BfCDCL^{L}$
8. $BfCDCL^{L}$ + Trypsin
9. $BfCDCL^{S}$
10. $BfCDCL^{S}$ + Trypsin
11. $BfCDCL^{L}$ + $BfCDCL^{S}$
12. $BfCDCL^{L}$ + $BfCDCL^{S}$ + Trypsin
13. PFO

Supplementary Fig. 5. *CDCL proteolytic activation sites.* Each CDCL (long and short) was digested with the indicated protease and analyzed via SDS-PAGE. Digests with protease of EaCDCL or BfCDCL were carried out at a 1:7 ratio (w/w) protease to CDCL for various times. The activated fragment was excised, and the amino terminal sequence was determined (Tufts University Core Facility for protein sequence analysis). Five cycles were conducted to sequence the N-terminal region of the activated fragment and the amino acids are listed.

Protease	Trypsin		Fragipain		Doripain B	
Cycle #	BfCDCL^{L}	BfCDCL^{S}	BfCDCL^{L}	BfCDCL^{S}	BfCDCL^{L}	BfCDCL^{S}
1	(**A**, S, G)	D, (**S**)	**A**	**S**	**A**	**S**
2	(**S**, Q, L, E)	(**S**, E), (V, I)	**S**, (**I**, P)	**S**, (V, I, A, F)	**S**	**S**, (I, R)
3	(**E**), (F)	**E**, V, H	**E**, (G, Y)	**E,** (D, K)	**E**	**E**
4	(**I**)	**L**, I	**I**, (K, A)	**L**, (K, **I**, M, T, Y)	**I**	**L**
5	(**I**)	**L**, S, (A, Y, P)	**I**, (P, D, Q)	**L**, (A, S, D, Q)	**I**	**L**
Cleavage Site	TR^{70}	TR^{62}	TR^{70}	TR^{62}	TR^{70}	TR^{62}

BfCDCL^{L}
MRILRYTLFLFAIGLISCEQEFDKKEQQSDCCSFQNLPKYPEQVVNINVHNYIPDMGDKVLSSPSHIH**TR**$_{70}$ASEIISETLW
DNGFGKTIHAQADESIVLDNLNRYIYPGSILDGASVANQDYKTISVHYKPINVSVSFPAQKVTGVLEKPSLSSCRQLVMDL
MHQKGIGQQSASVHFDIHRFTSYDELKMTFGSNANTSFLFWGSSSSQQEHKERISKSSGLYIRFIQKYFTIDMDIPEKSFI
EGSINAQYSPVYVSSIAYGRVGILTLETDEIYENAESIVKKAVNGFLYNKKEFLTVEEKGFFDEARMKVYVGGGNGDSGV
KTLTGFDDFIKFISEGGHFSAETPGKPIFCSFAYLSDHSPYKVKFKIDIDSDPVYARIEYRNLKNDSYVGRSLQREKIIGDV
HLAFYADRTAKIPTVAPRYISFNIVEHSRHYLKARIPRTEKDDTAVEEFVKSNNSRGTELQLKHDLLLTEFVWTHNNPRRT
RYEQNTFRYSLSEGGFYKILPAINEPKKWR

BfCDCL^{S}
MKQYVYLLILPIMFLISCSDDTVEEMGGSNHVLLERYPQEVINISMDNPESFASFNKTSV**TR**$_{62}$SSELLKDSVWSSGNGHT
LIYETNEHIPTQGLMRYIYLGSILQGGSIEKQRFVPIVKPMDPITISYSFPARWVTDIIAKPSLSAQRQSLQNIMNKEGMTG
KQLGSFTYNMRQFSYFEELKLAFGANVNIGGLLNIDVSLDKGKIRKKTGLFAKIVQRNYTVDMDLPADGNILLNHDDMGS
VGKYDPIYISSITYGRMALISIESSESYDKVRIALQAALQAKVVNGKLSFNLEQEKILKEAEVNVMVYNGEGEGTVKTAKG
WNEFQDFIIQGGRFSKDLPGDAIFYTASYLSDNSPFYSKFKIHLEN

Protease	Trypsin		Fragipain		Proteinase K	
Cycle #	EaCDCL^{L}	EaCDCL^{S}	EaCDCL^{L}	EaCDCL^{S}	EaCDCL^{L}	EaCDCL^{S}
1	**S**, (G, K, D)	**A**, (G)	**S**	**A**	**S**, (N, E)	**S**
2	**L**	**V**	**L**	**V**, (P)	**L**, (K)	**L**
3	**N**	**L**	**N**	**L**, (I, E)	**N**, **I**, F, V, (P, S)	**N**
4	**L**	**T**	**L**	**T**, (A)	K, **L**, A, (G)	**P**, (H)
5	**I**	(**T**)	**I**	**T**, (P, **I**, D)	**I**, E, T, D, F	**Y**
Cleavage Site	TK^{66}	TK^{87}	TK^{66}	TK^{87}	TK^{66}	IA^{78}

EaCDCL^{L}
MRIYSLFSLALAVTSSVIITSCATDNLPDRSSVTNNMARVELPVINITSFGTKPSFLNIQKSAS**TK**$_{66}$SLNLIAENSGDTETKE
FESSESVVLNHLNRYVFPGSLLMGNSIQDLNYKPVFASLNPITVSLSIPAINQNTAITITNPSLSATRAAVYNYLKTADFTQN
GQLSYSIQQFSSYDELKVAFGSNVNSRNLFGKNSSSTNVEEGMVARQSGFYVKFYQTSFTLDMDVPNGSLVKDNNFD
SEGIEPVYVSSISYGRMGILAIETNEKAEDAKRIINETFNKLFYKKQTNFSQEEKSFIEGADFNLYLVGGDGSTASQSFKG
YEAFVNHVSQGTFSKDQPGVPIFCSYSYLKDNSPVKTKFKFDIKRPPLYVKLVKENMKDINFNDPDGGIYDNKKEAILKIY
FYKNRSLVPTLPNPYINFKIREKKKKWQSIAPVYYSSLDQVPFNISERILTKQNTLQNIFATIQTQDNTEFSLISRIIRGGGR
QNPVPAGFRAIEINDYELVEDSNYIIIKD

EaCDCL^{S}
MKKLLFPIVALAATSLTIISCRQDSEVNPLQVQNSSKVLNPNVTLPANNLLYDEFFVSKESKLIEDSRNNKRKTSK**IA**$_{78}$SLN
PYAS**TK**$_{87}$AVLTTTSSTLTSDQIVVTVPQKTFIGGVYNSTTLDNLDYTPISYPLDPITVSYSFPSDFIVDTIERPSLSSMRASV
FKAMRAANFSGEQSLAFDYNIKQFSYYSELKIAFGSNVNIGKIFSIDISGSNNKIKRTTGVFAKFTQKNFTIDMDLPADGNI
FKNNSDLALTNGKNPVYISSVTYGRLGIISIESNASYNEVNFALKAALTAGIVNGSLNIDSNSKKILEESDLSVYLVGGRGT
DAVQVIKGFAGFSNFIVNGGQFTPEAPGVPIYFSASHASDNSVYYTTFTIDK

Supplementary Fig. 6. *Control samples for negative-stain EM imaging of activated EaCDCL samples.* (a, b) Representative micrographs of negative-stain grids prepared using activated-EaCDCL^{S} (0.3 µM) on lipid monolayers composed of POPC. (c-d) Representative micrographs of negative-stain grids prepared using gold labeled activated-EaCDCL^{L} (C347A/N239C mutant) (0.15 µM) on lipid monolayers composed of POPC. Electron micrographs are representative of at least three different grid squares. (E) Representative micrograph of negative-stain grids prepared gold labeled activated-EaCDCL^{L} (C347A/N239C mutant) (0.18 µM) in the absence of lipid.

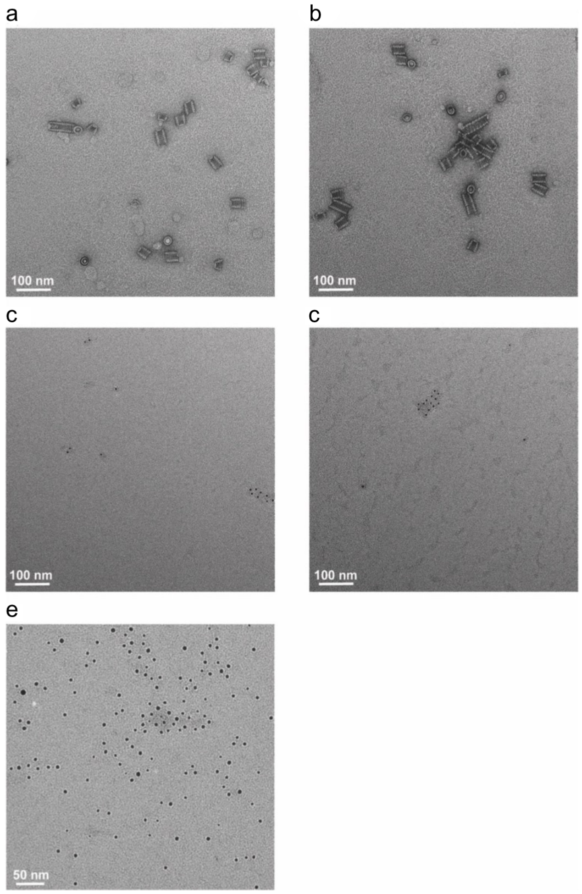

Supplementary Fig. 7. *$CDCL^L$ forms the membrane platform to assemble $CDCL^S$ into the pore.* (**a**) In the left panel is the kinetic release of CF marker from POPC and POPC-cholesterol (45/55 mol% POPC/cholesterol, respectively) liposomes by the $CDCL^L$-PFO(D4) chimera generated by fusing domains 1-3 (D1-3) of $EaCDCL^L$ (residues 23-368) with PFO domain 4 (D4, residues 389-500) when mixed with native $EaCDCL^S$. Only when the fusion protein is mixed with native $EaCDCL^S$ and proteolytically activated is pore forming activity only observed on the cholesterol-containing liposomes whereas the native EaCDCL pair is active on POPC membranes, as shown in Fig. 3. In the bottom panel pore formation on the POPC-cholesterol liposomes is only observed if the chimera is proteolytically activated. Note that the same emission profile for the proteolytically activated chimera is use in both graphs, as the experiments in both panels were carried out simultaneously. Conditions and protein concentrations were as described for the native CDCLs in the Methods section that describes the kinetic marker release assays from liposomes. (**b**) Hemolytic assays were carried out with PFO, the $EaCDCL^L$ and the $EaCDCL^L$-PFO^{D4} chimera with the wildtype $EaCDCL^S$. The starting concentrations of each protein is shown and was diluted 2-fold in 7 wells of a microtiter plate in triplicate in a volume of 150µl to which 50µl of washed sheep erythrocytes (3x in HBS) were added to make the final concentration of erythrocytes 5%. The plate was incubated at 37°C for 60 min and then spun at 4000xg to pellet the erythrocytes. 50µL of the supernatant from each well was transferred to a fresh microtiter plate and the hemoglobin content was determined at 540nm in a BMG Fluostar plate reader. UT, untreated sheep eryrthrocytes. Each hemolytic assay was done in triplicate and is shown on the bar graph.

a

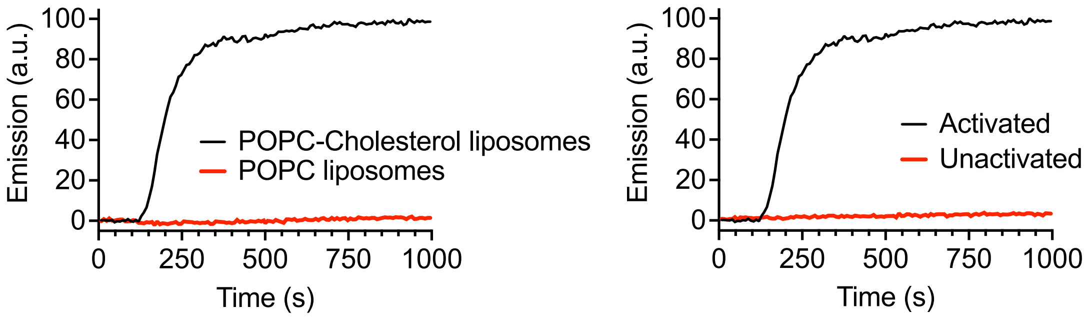

b

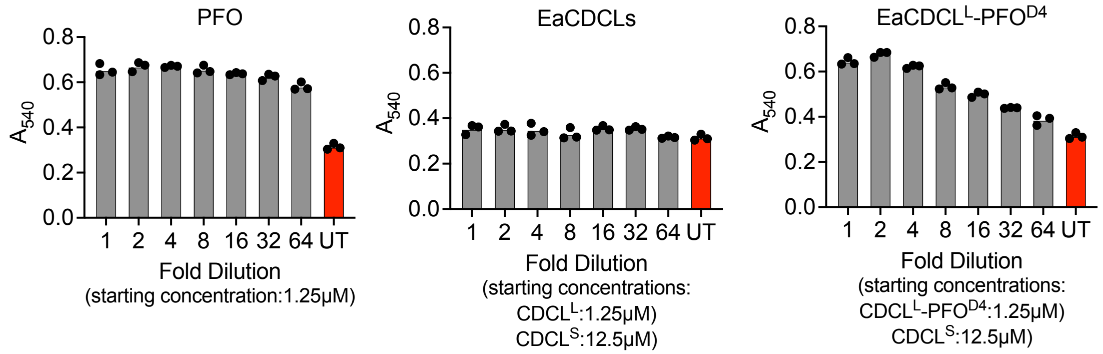

Supplementary Fig. 8. *Protein release from BfCDCL-treated P. dorei*. Based on the electron micrographs (Fig. 3c) the diameter of the CDCL pore is ~20nM, which is large enough for most proteins to pass through. Shown in (**a**) is a growth curve of *P. dorei* 9_1_42FAA of BfCDCL-untreated and treated cells. BfCDCL or HBS was introduced when the cells reach an OD_{600} of 0.3 (arrow) and then the cells were harvested at the end of the growth shown on the graph (~330 min). The culture media was separated from the cells and concentrated18-fold. The culture media was separated by SDS-PAGE (10% gel) (**b**) from the untreated and BfCDCL-treated *P. dorei* 9_1_42AA cultures. The bands subjected for mass spectroscopy are numbered. (**c**) The lanes outlined in the with the yellow boxes were subjected to densitometry using Image J. Spent media from untreated cells showed few clear proteins, whereas the treated cells exhibited many proteins (other than the BfCDCL proteins). When these protein bands (numbered) were subjected to mass spectrometry periplasmic proteins were identified, as well as several cytoplasmic proteins, particularly the stress-response chaperone DnaK (Supplementary Dataset 2).

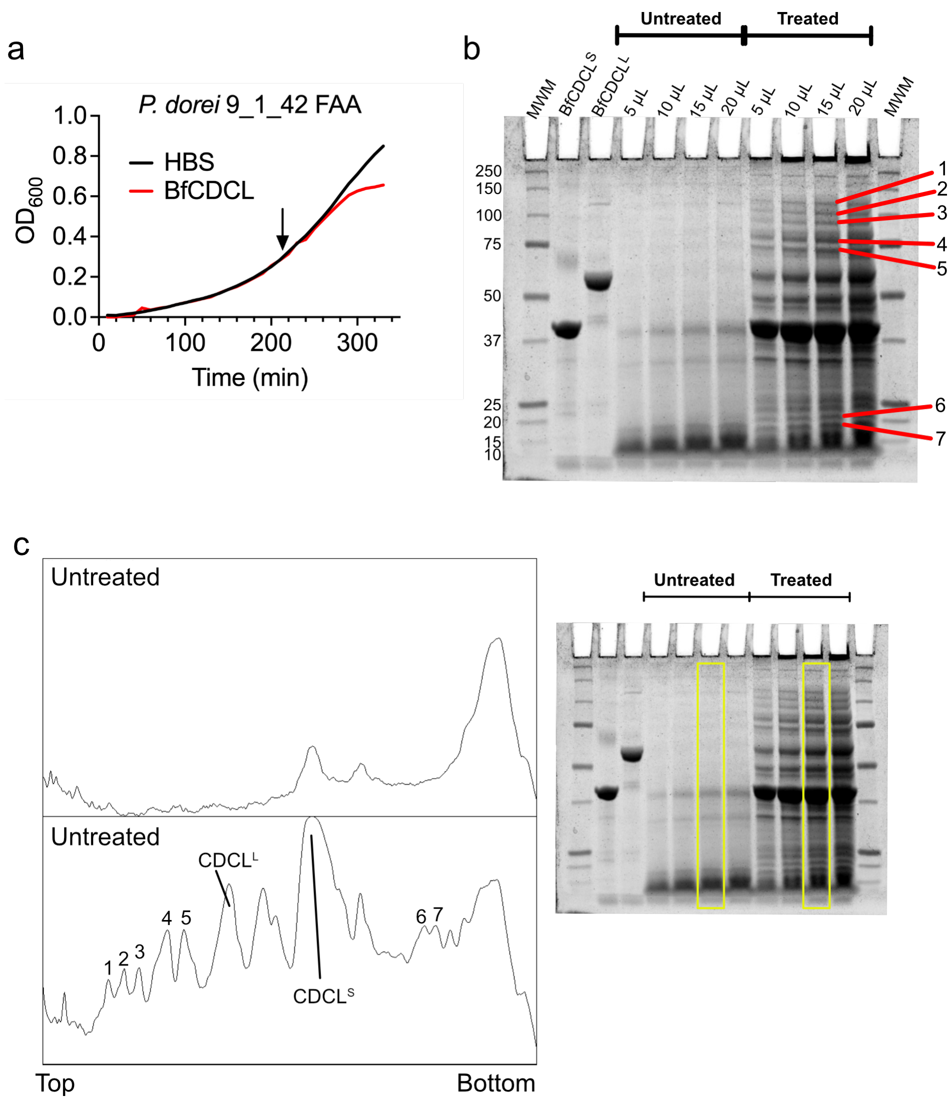

Supplementary Fig. 9. *Cleavage of BfCDCLs with the DpnB C11 protease.* Digest of BfCDCL^{L} and BfCDCL^{S} with purified DpnB were carried out in a 21.5uL reaction containing 4ug BfCDCL and the respective ratio (w/w) of DpnB indicated in each lane solubilized in HBS. Samples were incubated overnight at 37°C and quenched with 4x SDS loading buffer and DTT. All reactions were heated at 95°C for 5 minutes, analyzed via SDS-PAGE (10%), and stained with Coomassie Blue (see Methods for staininjg procedure). UL and US, unactivated BfCDCL^{L} and BfCDCL^{S}. AL and AS, activated BfCDCL^{L} and BfCDCL^{S} .Note that the His-tag is likely cleaved off by the DpnB protease in all cases and is responsible for the slight shift of the size of the treated wildtype and mutant proteins. Purified DpnB is autoprocessed into is not highly active in its purified form and is proteolytically activated during its expression in *E. coli*.

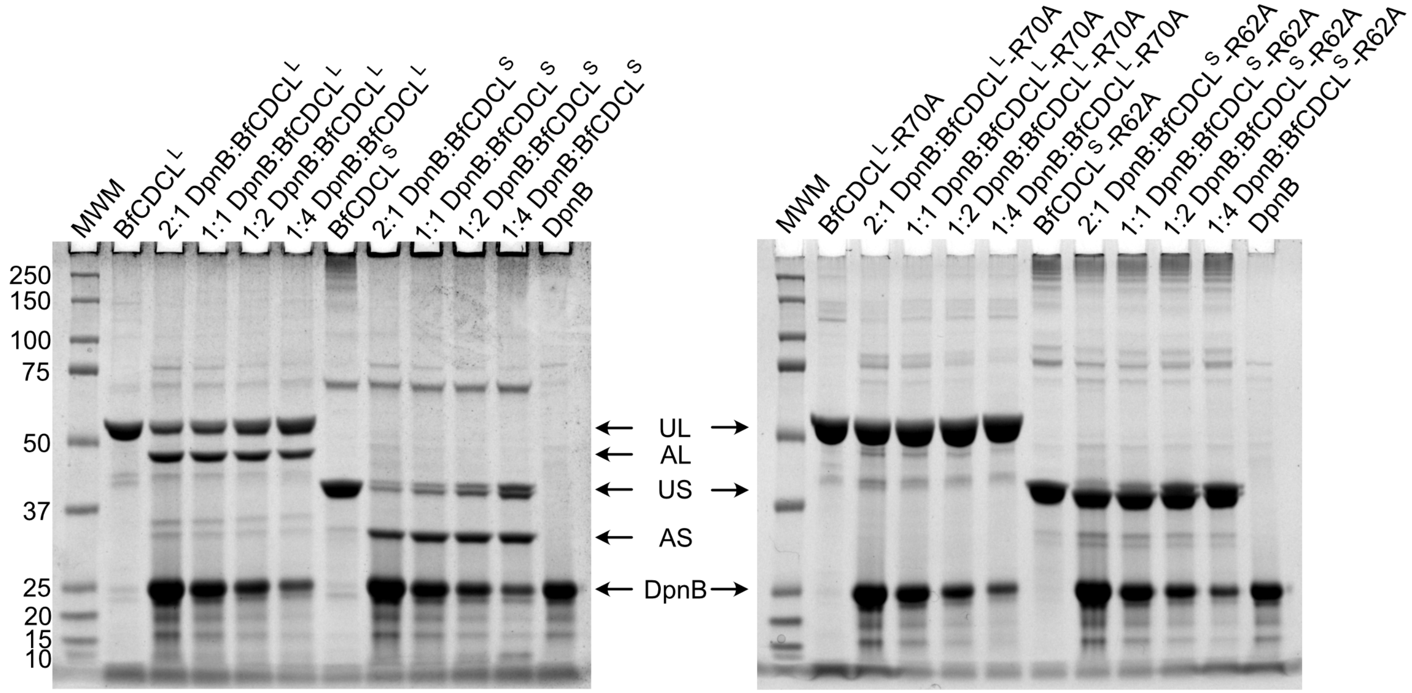

Supplemental Table 1: Data collection and refinement statistics for EaCDCL^S crystal structure.

**Data collection**	
Wavelength (Å)	0.9537 Å
Unit cell parameters:	
a, b, c (Å)	171.6, 171.6, 61.3
α, β, γ (°)	90, 90, 120
Space group	$P3_221$
Resolution range (Å)	74.32 - 1.85 (1.916 - 1.85)
Unique reflections	88,308 (8,759)
Completeness (%)	98.02 (92.83)
Multiplicity	29.7 (18.3)
Mean I/σ_I	13.61 (1.58)
$CC_{1/2}$	0.999 (0.623)
R_{pim}	0.03967 (0.5924)
Wilson B-factor ($Å^2$)	21.32
**Refinement**	
Reflections used in refinement	86577 (8142)
Reflections used for R_{free}	1950 (186)
R_{work}	0.1617 (0.2624)
R_{free}	0.1803 (0.2668)
No. non-hydrogen atoms	5474
macromolecules	5024
ligands	52
solvent	398
Protein residues	646
R.m.s deviations	
Bond lengths (Å)	0.012
Bond angles (°)	1.06
Ramachandran plot	
Favored (%)	98.43
Allowed (%)	1.57
Outliers (%)	0.00
Rotamer outliers (%)	0.87
Clashscore	2.68
Average B-factor ($Å^2$)	
macromolecules	32.49
ligands	32.11
solvent	56.29

Statistics for the highest-resolution shell are shown in parentheses.

Supplemental Table 2.

Purpose		Sequence
**Delete immunity gene (BF1274) from *B. fragilis* YCH46**	left flank forward	ggcatagtatcagatgagtggactacggaggcagtgttg
cloned into BamHI-digested pMLS36	left flank reverse	aagaggatcgtgctaaggaaacgaacagaag
	right flank forward	ttccttagcacgatcctcttataaacatgg
	right flank reverse	cgaattcctgcagcccggggccttcgcagttttaacag
**Construction of pKF35 integrative expression vector** (encoding erythromycin resistance)		
amplify pFD340 promoter region	forward	cggccgctctagaactagtgGGTCTGGATACGCAAGTTG
	reverse	cgaattcctgcagcccggggATCCTCTAGAGTCAATTCTGATTAATAATTTG
**Construction of pKF54 integrative expression vector (encoding cefoxitin resistance)**		
amplify *cfxA* from *P. vulgatus* CL11T00C01	forward	gatgaaccatgagtacgttcggatacgtccgtgtcaactc
	reverse	atttacagttgcatgtggccgccgactggttcagggag
amplify pKF35 without *ermG*	forward	ggccacatgcaactgtaaatg
	reverse	gaacgtactcatggttcatc
**Clone immunity gene (Bf1274) for expression (into pKF35 or pKF54)**	forward	aatcagaattgactctagaggcaaaaaactaaactgtgttaatatg
	reverse	tcgaattcctgcagcccggggttaaaaatcaaaagtaccatgtttataag

Supplemental Table 3. *Bacterial strains and plasmids used in this study*. Shown are the bacterial strains and their derivatives used herein and the plasmids carrying the various recombinant wildtype CDCL, Dpn proteases and immunity (BcIP) proteins and their derivatives.

Strain	Source or reference
One Shot™ BL21(DE3) Chemically Competent *Escherichia coli*	Invitrogen
Bacteroides fragilis YCH46	reference[3]
Bacteroides fragilis YCH46 Δ*bcdI*	This study
Bacteroides fragilis 638R	Lab strain
Bacteroides ovatus ATCC 8483	ATCC
Bacteroides uniformis ATCC 8492	ATCC
Bacteroides nordii CL02T12C05	reference[4]
Phocaeicola vulgatus ATCC 8483	ATCC
Phocaeicola vulgatus CL10T00C06	reference[4]
Phocaeicola vulgatus CL09T03C04	reference[4]
P. vulgatus CL09T03C04 pKF54 (vector)	This study
P. vulgatus CL09T03C04 p*bcdI*	This study
Phocaeicola dorei CL03T12C01	reference[4]
Phocaeicola dorei 5_1_36/D4	BEI
Phocaeicola dorei 9_1_42FAA	BEI
P. dorei 9_1_42FAA pKF35 (vector)	This study
P. dorei 9_1_42FAA p*bcdI*	This study
Phocaeicola dorei CL02T00C15	reference[4]
P. dorei CL02T00C15 Δ*dpnA*	reference[5]
P. dorei CL02T00C15 Δ*dpnB*	reference[5]
$EaCDCL^L$ (pET-15b)	This study/Genscript
$EaCDCL^S$ (pET-15b)	This study/Genscript
$EaCDCL^L$-K66A (pET-15b)	This study/Genscript
$EaCDCL^S$-K87A (pET-15b)	This study/Genscript

EaCDCL^{L}-C347A, N239C (pET-15b)	This study/Genscript
EaCDCL^{S}-N248C (pET-15b)	This study/Genscript
BfCDCL^{L} (pET-15b)	This study/Genscript
BfCDCL^{S} (pET-15b)	This study/Genscript
BfCDCL^{L}-R70A (pET-15b)	This study/Genscript
BfCDCL^{S}-R62A (pET-15b)	This study/Genscript
BfCDCL^{L}-C31S, C32S, S245C (pET-15b)	This study/Genscript
BfCDCL^{S}-K361C (pET-15b)	This study/Genscript
BfIP (pET-15b)	This study/Genscript
Doripain A (pET-15b)	This study/Genscript
Doripain B (pET-15b)	This study/Genscript
Fragipain (pET-15b)	This study/Genscript
EaCDCL^{L} domains 1-3 fusion to PFO domain (pET15b)	This study/Genscript

Supplementary References

1. Sievers, F., Wilm, A., Dineen, D., Gibson, T.J., Karplus, K., Li, W., Lopez, R., McWilliam, H., Remmert, M., Soding, J., et al. (2011). Fast, scalable generation of high-quality protein multiple sequence alignments using Clustal Omega. Mol. Syst. Biol. *7*, 539. 10.1038/msb.2011.75.
2. Shepard, L.A., Shatursky, O., Johnson, A.E., and Tweten, R.K. (2000). The mechanism of assembly and insertion of the membrane complex of the cholesterol-dependent cytolysin perfringolysin O: Formation of a large prepore complex. Biochemistry *39*, 10284-10293.
3. Kuwahara, T., Yamashita, A., Hirakawa, H., Nakayama, H., Toh, H., Okada, N., Kuhara, S., Hattori, M., Hayashi, T., and Ohnishi, Y. (2004). Genomic analysis of *Bacteroides fragilis* reveals extensive DNA inversions regulating cell surface adaptation. Proc. Natl. Acad. Sci. U. S. A. *101*, 14919-14924. 10.1073/pnas.0404172101.
4. Zitomersky, N.L., Coyne, M.J., and Comstock, L.E. (2011). Longitudinal analysis of the prevalence, maintenance, and IgA response to species of the order Bacteroidales in the human gut. Infect Immun *79*, 2012-2020. 10.1128/IAI.01348-10.
5. Evans, J.C., McEneany, V.L., Coyne, M.J., Caldwell, E.P., Sheahan, M.L., Von, S.S., Coyne, E.M., Tweten, R.K., and Comstock, L.E. (2022). A proteolytically activated antimicrobial toxin encoded on a mobile plasmid of Bacteroidales induces a protective response. Nat Commun *13*, 4258. 10.1038/s41467-022-31925-w.
