# Supplementary material for: Distant relatives of a eukaryotic cell-specific toxin family evolved a complement-like mechanism to kill bacteria
Source: Nat Commun. 2024 Jun 12;15:5028. doi: 10.1038/s41467-024-49103-5 (PMC11169675; doi:10.1038/s41467-024-49103-5)
Supplement: Supplementary file 3 — Description of Additional Supplementary Files [file 41467_2024_49103_MOESM3_ESM.pdf]

Description of Additional Supplementary Files:

Supplementary Dataset 1: Prevalence of CDCL proteins, including similarity matrices and grouping by pattern analyses present in sequenced bacterial genomes.

Supplementary Dataset 2: This dataset shows the prevalence of CDCL genes in human gut metagenomic datasets. Sixteen human gut metagenomic datasets were analyzed comprising 1958 subjects.

Supplementary Dataset 3: This dataset shows the top mass spec hits for the proteins released by treatment of P. dorei 9_1_42FAA with the Bacteroides fragilis CDCL.
